# Postoperative leukopenia after cytoreductive surgery and hypertherm intraperitoneal chemotherapy for colorectal carcinomatosis– causes and implication on outcomes in a population-based study

**DOI:** 10.1186/s12957-025-03821-2

**Published:** 2025-04-29

**Authors:** Mattias Lepsenyi, Valentinus Valdimarsson, Nader Algethami, Henrik Thorlacius, Lana Ghanipour, Peter Cashin, Dan Asplund, Elinor Bexe Lindskog, Gabriella Jansson Palmer, Per J Nilsson, Ingvar Syk

**Affiliations:** 1 Department of Clinical Sciences Malmö, Section of Surgery, Lund University, Skåne University Hospital, Inga Marie Nilssons gata 47, Malmö, 20502 Sweden; 2 Department of Surgical Sciences, Section of Surgery, Uppsala University, Uppsala, Akademiska sjukhuset, Sweden; 3 Department of Surgery, Institute of Clinical Sciences, Sahlgrenska Academy, University of Gothenburg, Gothenburg, Sweden; 4 Region Västra Götaland, dept of Surgery, Sahlgrenska University Hospital, Gothenburg, Sweden; 5 Department of Pelvic cancer, GI Oncology and Colorectal Surgery Unit, Karolinska University Hospital, Stockholm, Sweden; 6 Department of Molecular Medicine and Surgery, Karolinska Institutet, Stockholm, Sweden

**Keywords:** Cytoreductive surgery, Hyperthermic perioperative chemotherapy, Postoperative leukopenia, Postoperative complication

## Abstract

**Background:**

Leukocytes have been reported to have tumor stimulating effects in colorectal cancer, among other malignancies. In line with this, earlier research has shown improved disease-free survival in patients with postoperative neutropenia compared to non-neutropenic patients following cytoreductive surgery (CRS) and hypertherm intraperitoneal chemotherapy (HIPEC).

**Aim:**

To evaluate the impact of postoperative leukopenia after CRS and HIPEC on recurrence rate, survival, and risk of complications.

**Methods:**

All CRS and HIPEC-procedures for colorectal adenocarcinoma in the national Swedish HIPEC-registry since 2015 and local registries in Uppsala and Malmö since 2003 until December 31st, 2021, were included (*n* = 921). Patients who did not complete a full CRS and HIPEC procedure (*n* = 99), had incomplete macroscopic cytoreduction (*n* = 25) or a lack of information on leukocyte count (*n* = 213) were excluded, resulting in 584 analyzed cases. Primary outcome was overall recurrence rate. Secondary outcomes were overall survival, recurrence-free survival, and perioperative complications.

**Results:**

Postoperative leukopenia was observed in 54 (9.2%) cases of which 32 (5.5%) developed severe leukopenia. No differences in patient characteristics were noted between those with or without leukopenia. There were no differences in 3-year recurrence rate, overall survival or 3-year recurrence-free survival, between the groups. Neoadjuvant chemotherapy treatment, HR 1.32 (95% CI: 1.02–1.71), higher PCI-score, HR 1.50 (95% CI: 1.09–2.05) and higher pN-stage HR 2.52 (95% CI: 1.74–3.65) were associated with higher 3-year recurrence rate. 3-year mortality was associated with neoadjuvant chemotherapy treatment, HR 1.82 (95% CI: 1.06–3.11), severe postoperative complication, HR 2.39 (95% CI: 1.39–4.13) and high PCI-score, HR 2.60 (95% CI: 1.31–5.14). Treatment with combined oxaliplatin/irinotecan, HR 12.34 (95% CI: 4.51–33.74) was associated with developing postoperative leukopenia. Longer operation time, HR 2.30 (95% CI: 1.55–3.42), and severe leukopenia, HR 3.50 (95% CI: 1.25–9.77) were associated with postoperative complication.

**Conclusions:**

Postoperative leukopenia did not impact recurrence rate or long-term survival in a statistically significant manner. Neoadjuvant chemotherapy and high PCI-score were associated with both recurrent disease and mortality within 3 years.

**Supplementary Information:**

The online version contains supplementary material available at 10.1186/s12957-025-03821-2.

## Introduction

Peritoneal carcinomatosis (PC) is present in 5 to 10% of patients with colorectal adenocarcinoma as synchronous metastases and in 30 to 40% of patients with metachronous spread [1, 2]. These patients have historically been considered palliative, with a median survival of 5 to 13 months depending on whether systemic chemotherapy is given or not [3, 4]. In the last decades, the introduction of cytoreductive surgery (CRS) and perioperative intraperitoneal (ip) chemotherapy, administrated as early postoperative chemotherapy (EPIC) or hypertherm intraperitoneal chemotherapy (HIPEC), has transformed PC into a potentially curable situation in selected patients, with 5-year survival rates ranging from 30 to 50% [3, 5, 6]. Low tumor burden, favorable tumor biology, good performance status, and absence of serious comorbidity are associated with an improved long-term recurrence-free survival [7, 8].

Recent research suggests that postoperative immune suppression after cancer surgery could have a beneficial effect on recurrence and survival [9]. One plausible underlying mechanism is that activated neutrophils in areas of inflammation and wound healing expel nucleic DNA in web-like structures known as neutrophil extracellular traps (NETs). These NETs are covered with cytoplasmatic proteins such as elastase and citrullinated histones, and act by binding pathogens for elimination by the immune system [10]. Findings the last few years show that tumor cells utilize NETs for adhesion, migration and growth while evading host immune cells. By reducing neutrophil count, a statistically significant reduction of NETs in the tissue, and consequently less adhered tumor nodules, has been experimentally demonstrated [11]. A previous study has shown that postoperative neutropenia following CRS and HIPEC, for colorectal cancer, was associated with improved disease-free survival [12].

Based on the findings described above, this study aimed to further investigate a possible impact of postoperative immune suppression measured as leukocyte count on long-term results after CRS and HIPEC. The hypothesis was that postoperative leukopenia after CRS and HIPEC for peritoneal spread of adenocarcinoma of colorectal origin would have a positive effect on recurrence rate, disease-free survival and overall survival.

## Materials and methods

### Study population

The implementation of CRS and EPIC/HIPEC in Sweden began at Uppsala University Hospital in 2003 and Malmö/Skane University Hospital in 2004. From the start, both centers implemented local registries for all treated patients with prospectively collected data. Following the introduction of CRS and HIPEC in Stockholm and Gothenburg, a national HIPEC registry was established in 2015. Since then, all patients undergoing CRS and HIPEC in Sweden have been prospectively enrolled in the national registry.

This study encompasses all patients in both the local and national HIPEC registries from January 2003 to December 2021, thus including all patients treated in Sweden during this period. Patients with confirmed colorectal adenocarcinoma or goblet cell carcinoma in the appendix, colon or rectum who received CRS and HIPEC were included. Cases where information was lacking on postoperative leukopenia, not completing a full CRS and HIPEC procedure or cases not achieving clinical complete macroscopic cytoreduction (CC ≠ 0) were excluded. In patients that have undergone re-HIPEC, only the first event was factored into the survival analyses.

### Outcomes and definitions

The primary outcome was recurrence rate. Secondary outcomes were overall survival, recurrence-free survival, time to recurrence, and perioperative complications.

Leukopenia was defined as white blood cell count (WBC) < 1.6 × 10⁹/L and severe leukopenia as WBC < 1.0 × 10⁹/L. Tumor burden, measured as PCI-score, was grouped in three levels: <8, 9–15 and > 15. Survival as well as time to recurrence was calculated starting at the date of surgery. Recurrence was defined as clinical signs of recurrent disease, usually based on radiologic imaging, with or without histopathological diagnosis. The Clavien-Dindo (CD) score [13] was used for the classification of postoperative complications. Only the most severe complication was registered in each patient. A score of CD grade 3b or higher, indicating the need for intervention under general anesthesia, was defined as severe complication. Leukopenia as registered complication was excluded in the complication analyses. In long-term survival analysis, mortality within 90 days postoperatively was excluded, to evaluate the long-term effect of leukopenia more specifically.

### Statistical analysis

Continuous variables are presented as medians with interquartile ranges (IQR) and group comparisons were conducted using the Mann-Whitney U-test. Categorical variables are presented as proportions and group comparisons were made using the Chi-square test. The Cox proportional hazard ratio model and logistic regression were used for the multivariate analyses. All variables which differed between the groups in univariate analyses with a p-value < 0.20 were included in the multivariate analyses. To test the robustness of this model, sensitivity analyses were performed.

The Kaplan-Meier method was used for survival estimations of median overall survival (OS) and recurrence-free survival (RFS). The Log-Rank test was used for group comparisons.

Two-sided *p*-values lower than 0.05 were considered statistically significant. Data was analyzed using SPSS (statistical package for social sciences, IBM Corporation Armonk, NY, USA, version 28.0.0.0). The study was approved by the Swedish Ethical Review Authority, 2020/03504.

## Results

A total of 921 colorectal cancer cases were identified in the registries. Of these, 213 were excluded due to missing information on leukopenia (predominantly before 2009), 99 were excluded as they did not undergo complete CRS and HIPEC (CRS only or open and close procedures). Another 25 cases were excluded due to incomplete macroscopic cytoreduction (CC ≠ 0). Hence, 584 patients were finally included in the study (Suppl Fig. 1). Of these, 187 had metachronous PC and 347 synchronous PC (missing data = 50). Ten patients with synchronous PC had surgery for the primary colorectal tumor prior to CRS and HIPEC.

A total of 54 (9.2%) cases developed postoperative leukopenia, of which 32 (5.5%) were severe. The leukopenia and non-leukopenia groups did not differ statistically significant, in any patient characteristics (Table 1).

### Primary outcome

**Fig. 1:**
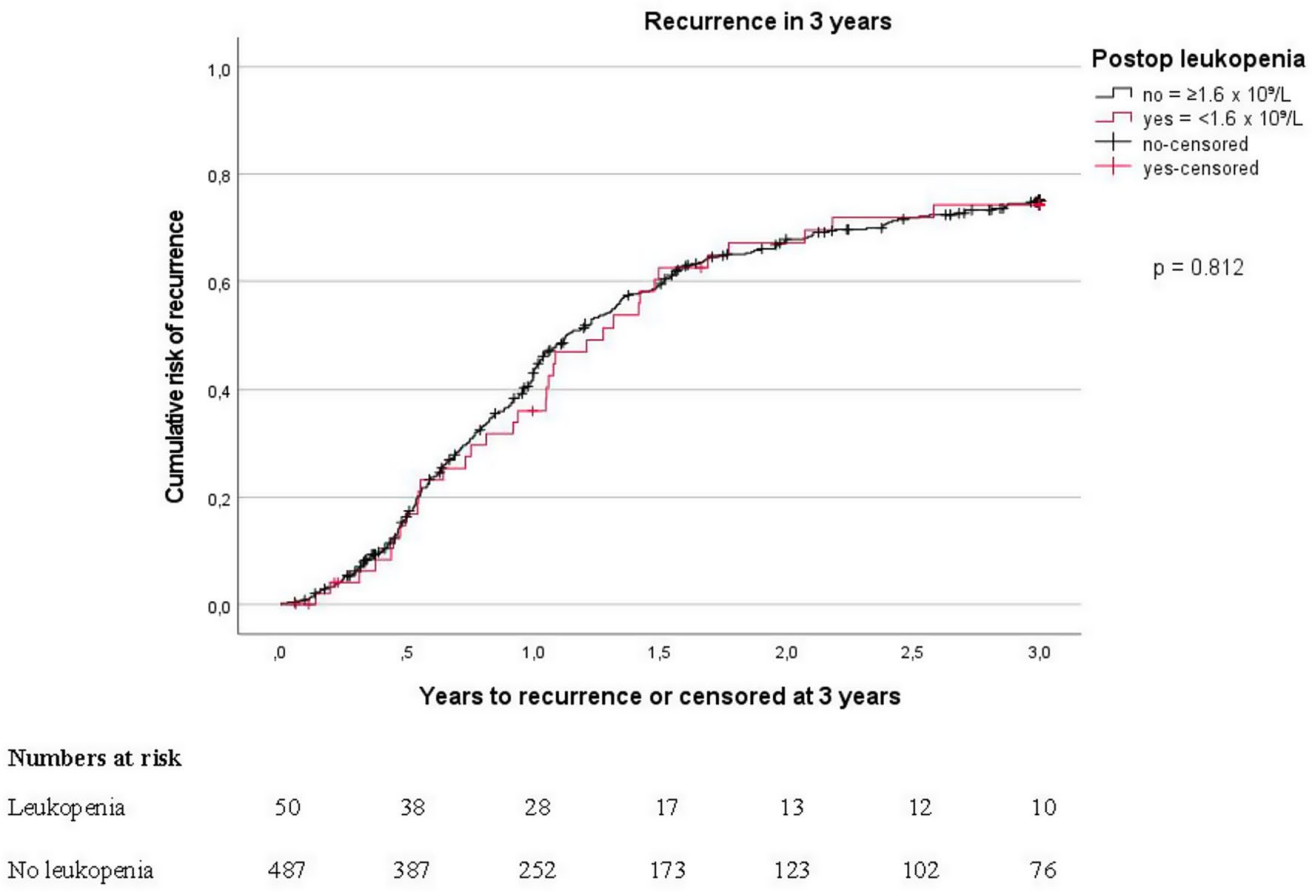
3-year recurrence rate stratified on leukopenia and no leukopenia, 90-day mortality excluded from analysis

The overall 3-year recurrence rate was 75.1%, without statistically significant difference between the leukopenia or severe leukopenia groups compared to the non-leukopenia group (Fig. 1, Table 2A and B). In multivariate analyses of risk of recurrence, neoadjuvant chemotherapy treatment, HR 1.32 (95% CI: 1.02–1.71) was associated to increased recurrence rate, as was higher pN-stage, HR 2.52 (95% CI: 1.74–3.65) and higher PCI-score, HR 1.50 (95% CI: 1.09–2.05) (Table 3), whereas leukopenia did not affect the risk of recurrence. The results were stable when tested in sensitivity analysis, (Supplementary Table 1).

### Secondary outcomes

**Fig. 2:**
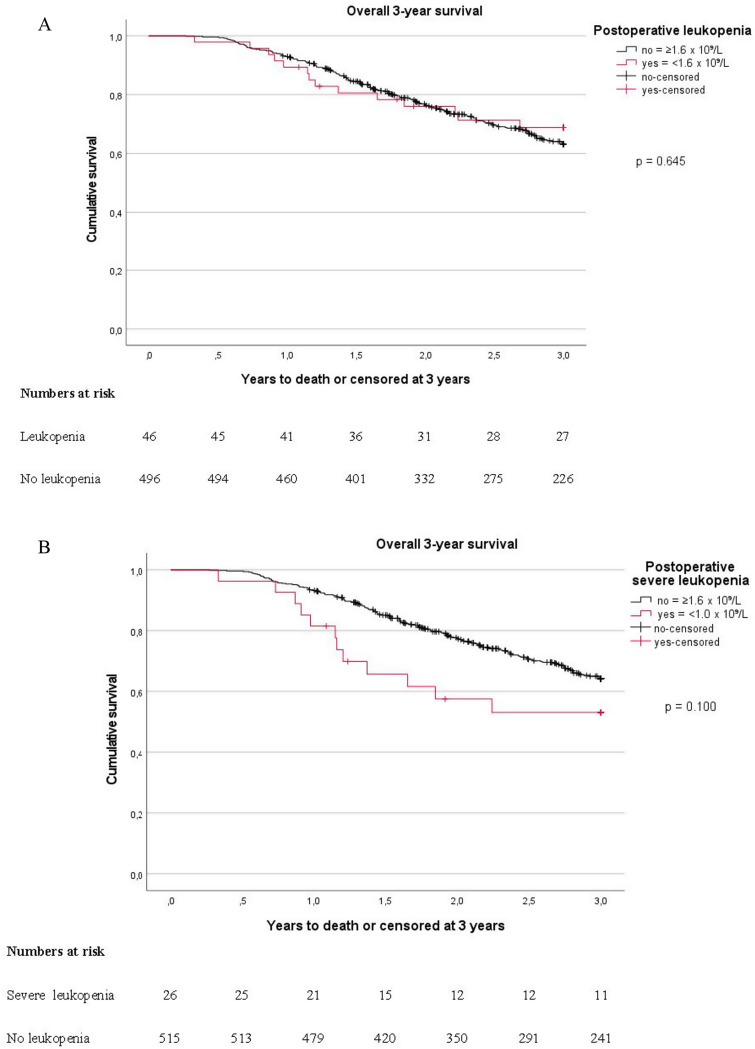
Three-year overall survival, 90-day mortality excluded from analysis. A, stratified on leukopenia and no leukopenia. B, stratified on severe leukopenia and no leukopenia

No difference in overall 3-year survival or 3-year recurrence-free survival was noted between the leukopenia and non-leukopenia groups, although the subgroup with severe leukopenia showed a tendency towards worse 3-year overall survival, 53.1% (95% CI: 36.7–76.8) compared to 63.4% (95% CI: 59.0-68.1), albeit statistically non-significant (Table 2; Fig. 2A and B). In multivariate analysis, neoadjuvant chemotherapy HR 1.82 (95% CI: 1.06–3.11), severe postoperative complication HR 2.39 (95% CI: 1.39–4.13) and higher PCI-score, HR 2.60 (95% CI: 1.31–5.14) were noted to be associated with of lower 3-year survival, (Table 4). The results were stable when tested in the sensitivity analysis, (Supplementary Table 2).

There was a statistically significant higher ratio of leukopenia in the group treated with combined ip irinotecan and oxaliplatin, compared to the group treated with oxaliplatin as single drug (45.9% vs. 6.1%, *p* = < 0.01), as well as treatment with mitomycin C versus oxaliplatin (17.2%, *p* = 0.026) (Table 5). The combination therapy was also associated with leukopenia in multivariate analysis, HR 12.34 (95% CI: 4.51–33.74), as was Mitomycin C, HR 3.00 (95% CI: 1.02–8.84), (Suppl. Table 3). Cases with operating time over the median (≥ 480 min) developed postoperative leukopenia in a higher ratio, (66.7% vs. 46.2%, *p* = 0.004) (Table 5). This finding could however not be confirmed in the multivariate analysis (Supplementary Table 3).

In multivariate analysis, operating time exceeding 480 min was associated with postoperative complications, HR 2.30 (95% CI: 1.55–3.42), as was severe leukopenia HR 3.50 (95% CI: 1.25–9.77) (Supplementary Table 4). Both were also associated with severe complications (Supplementary Table 5).

**Table 1:** Patient characteristics stratified on cases with postoperative leukopenia or no leukopenia

	Leukopenia	No leukopenia	Total
**Cases**, n (%)	54 (9,2)	530 (90,8)	584 (100)
**Age in years**, median	63.5	63	63
IQR	(57.4–69.3)	(51.7–70.0)	(52.0–70.0)
Missing	0	4	4
**Male/Female**, n	22/32	241/287	263/319
%	41	45.5	45.2
Missing data	1	1	2
**Histology**:			
Adenocarcinoma, n (%)	51 (94,4)	504 (95,1)	555
Gobletcellcarcinoma, n (%)	3 (5,6)	26 (4,9)	29
Missing data	0	0	0
**CEA**, g/L, median (IQR)	5 (2–30)	5 (2–16)	5 (2–17)
Missing data	10	45	55
**PCI-score**, median (IQR)	9 (6,5–16)	9 (4–15)	9 (4,25 − 15)
0–8, n (%)	26 (49,1)	253 (48,2)	279
9–15, n (%)	13 (24,5)	159 (30,3)	172
> 15, n (%)	14 (26,4)	113 (21,5)	127
Missing data	1	5	6
**Localization primary tumor**, n (%)			
Appendix, n (%)	5 (9,3)	36 (6,8)	41 (7,0)
Right colon, n (%)	21 (38,9)	206 (39,0)	227 (39,0)
Transvers colon, n (%)	5 (9,3)	44 (8,3)	49 (8,4)
Left/Sigmoid colon, n (%)	14 (25,9)	180 (34,1)	194 (33,3)
Rectum, n (%)	9 (16,7)	62 (11,7)	71 (12,2)
Missing data	0	2	2

**Table 2a:** Three-year recurrence rate and survival stratified on leukopenia and no leukopenia

	Missing data, *n*	Leukopenia (*n* = 54)	No Leukopenia (*n* = 530)	Total (*n* = 584)	
3-year recurrence, % (95% CI)	44	74.2 (57.6–84.3)	75.2 (70.6–79.1)	75.1 (70.8–78.8)	
Time to recurrence, median months	44	12.9	12.3	12.4	
3-year overall survival*, % (95% CI)	62	67.4 (54.6–83.1)	62.6 (58.1–67.4)	62.9 (57.8–67.5)	
3-year recurrence free survival*, % (95% CI)	47	22.9 (13.6–38.3)	17.7 (14.6–21.4)	18.1 (15.1–21.7)	

* 90 day mortality excluded (*n* = 12)

**Table 2b:** Three-year recurrence rate and survival stratified on severe leukopenia and no leukopenia

	Missing data (*n*)	Severe leukopenia (*n* = 32)	No leukopenia (*n* = 530)
3-year recurrence, % (95% CI)	51	74.4 (48.3–83.0)	75.1 (70.6–79.0)
Time to recurrence, median months	44	11.9	12.4
3-year overall survival*, % (95% CI)	69	53.1 (36.7–76.8)	63.4 (59.0-68.1)
3-year recurrence free survival*, % (95% CI)	54	28.7 (16.5–51.1)	17.8 (14.8–21.5)

* 90 day mortality excluded (*n* = 12)

**Table 3:** Risk of recurrence within 3 years* estimated by Cox proportional hazard ratio model

	Missing	In analysis,	Univariate analysis	*p*-value	Multivariate analysis	*p*-value
	data, *n*	*n*	HR (95% CI)		HR (95% CI)	
**Age**:	48					
< 65 years		292	ref.			
≥ 65 years		244	1.039 (0.846–1.276)	0.718		
**Sex**:	46					
Male		246	1.036 (0.844–1.273)	0.739		
Female		292	ref.			
**Neoadjuvant chemo**:	166					
Yes		125	1.323 (1.031–1.699)	0.028	1.319 (1.019–1.708)	0.036
No		293	ref.			
**Any complication**:	51					
Yes		305	1.081 (0.877–1.333)	0.464		
No		228	ref.			
**Severe complication**:	34					
Yes		68	1.037 (0.765–1.406)	0.814		
No complication		228	ref.			
Not in analysis (C-D 1-3a)		254				
**Duration of surgery**:	64					
< 480 min		271	ref.			
≥ 480 min		249	1.117 (0.910–1.370)	0.291		
**Postop leukopenia**:	44					
Yes		51	0.962 (0.676–1.369)	0.83	0.890 (0.586–1.351)	0.585
No		489	ref.			
**Severe leukopenia**:	43					
Yes		30	0.953 (0.670–1.356)	0.789		
No		489	ref.			
Not in analysis (mild leukopenia)		22				
**PCI-score**:	48					
0–8		265	ref.		ref.	
9–15		157	1.664 (1.310–2.115)	< 0.001	1.452 (1.096–1.922)	0.09
> 15		114	1.609 (1.242–2.084)	< 0.001	1.497 (1.093–2.050)	0.012
**pN-stage**:	68					
N0		106	ref.		ref.	
N1		183	1.961 (1.421–2.704)	< 0.001	1.908 (1.300-2.799)	< 0.001
N2		224	2.285 (1.672–3.123)	< 0.001	2.523 (1.743–3.653)	< 0.001
Nx		3				
**Period of surgery**:	44					
2019–2021		198	ref.		ref.	
2016–2018		221	0.881 (0.698–1.112)	0.287	0.906 (0.678–1.210)	0.504
2013–2015		100	0.726 (0.542–0.972)	0.031	0.680 (0.478–0.969)	0.033
≤ 2012		21	0.515 (0.262–1.112)	0.055	0.282 (0.111–0.714)	0.008

* 90 day mortality excluded (*n* = 12)

**Table 4:** Risk of mortality within 3 years* estimated by Cox proportional hazard ratio model

	Missing	In analysis,	Univariate analysis	*p*-value	Multivariate analysis	*p*-value
	data, n	n	HR (95% CI)		HR (95% CI)	
**Age**:	43					
< 65 years		299	ref.			
≥ 65 years		242	1.037 (0.773–1.391)	0.809		
**Sex**:	42					
Male		249	1.043 (0.777–1.399)	0.78		
Female		293	ref.			
**Neoadjuvant chemo**:	164					
Yes		127	1.376 (0.978–1.934)	0.067	1.815 (1.059–3.110)	0.03
No		293	ref.			
**Any complication** :	48					
Yes		311	1.663 (1.205–2.295)	0,002		
No		225	ref.			
**Severe complication** :	33					
Yes		72	2.112 (1.373–3.249)	< 0.001	2.390 (1.385–4.127)	0.002
No		225	ref.			
Not in analysis (CD 1-3a)		254				
**Duration of surgery**:	60					
< 480 min		267	ref.			
≥ 480 min		257	1.313 (0.975–1.769)	0.073	0.652 (0.382–1.113)	0.117
**Postop leukopenia**:	40					
Yes		47	0.877 (0.508–1.514)	0.638		
No		497	ref.			
**Severe leukopenia**:	38					
Yes		27	1.678 (0.934–3.015)	0.083	1.357 (0.448–4.111)	0.589
No		497	ref.			
Not in analysis (mild leukopenia)		22				
**PCI-score**:	45					
0–8		265	ref.		ref.	
9–15		159	2.084 (1.464–2.965)	< 0.001	2.318 (1.239–4.335)	0.009
> 15		115	2.632 (1.822–3.801)	< 0.001	2.598 (1.313–5.141)	0.006
**pN-stage**:	66					
N0		107	ref.		ref.	
N1		181	1.902 (1.118–3.234)	0.018	0.915 (0.392–2.138)	0.837
N2		227	2.991 (1.809–4.944)	< 0.001	1.844 (0.866–3.927)	0.113
Nx		3				
**Period of surgery**:	40					
2019–2021		201	ref.		ref.	
2016–2018		213	1.546 (1.070–2.234)	0.02	1.487 (0.748–2.954)	0.257
2013–2015		109	1.541 (1.008–2.355)	0.046	1.019 (0.467–2.226)	0.962
≤ 2012		21	1.425 (0.669–3.034)	0.358	0.842 (0.210–3.368)	0.808

* 90 day mortality excluded (*n* = 12)

**Table 5:** Rate of leukopenia depending on perioperative factors

	Missing, *n*	Leukopenia	No leukopenia	Total (*n*)	*p*-value
**Neoadjuvant therapy**: n (%)	131				
Yes		15 (11.0)	121 (89.0)	136	0.535
No		29 (9.1)	288 (90.9)	317	ref.
**Chemotherapy used in HIPEC**:	47				
Oxaliplatin, n (%)		24 (6.1)	371 (93.9)	395	ref.
Oxaliplatin + Irinotecan, n (%)		17 (45.9)	20 (54.1)	37	< 0.01
Mitomycin C, n (%)		5 (17.2)	24 (82.8)	29	0.026
Irinotecan, n (%)		5 (7.1)	65 (92.9)	70	0.719
Other, n (%)		2 (33.3)	4 (66.7)	6	-
Missing data, n (%)		1 (2.1)	46 (97.9)	47	-
**Operating time**:	0				
< 480 min		18 (33.3)	285 (53.8)	303	ref.
≥ 480 min		36 (66.7)	245 (46.2)	281	0.004

## Discussion

This study did not show any differences in recurrence rate or survival after CRS and HIPEC for peritoneal carcinomatosis of colorectal cancer, in patients who developed postoperative leukopenia compared to those who did not. On the contrary, the subgroup with severe leukopenia showed a tendency towards worse three-year overall survival compared to the non-leukopenia group. These findings contrast with a previous study by Cashin et al. [12], who reported a statistically significant higher disease-free survival in the group with postoperative neutropenia compared to non-neutropen patients following CRS and HIPEC. In that study, 246 HIPEC-procedures from a merged dataset of Uppsala, Sweden and St Georges hospital in Sydney, Australia, also showed a tendency towards better overall survival in the neutropenia group, albeit not statistically significant.

Although the finding in the study by Cashin et al. of improved recurrence rate in the neutropenia group was incidental, it supports the hypothesis of a tumor stimulating effect of leukocytes. A theoretical explanation for this seemingly contradictive effect might be related to the immune response. There is increasing evidence on the interaction of immune cells and tumor cells, leading to stimulated migration, adhesion, and growth of tumor cells [9, 14, 15]. Specifically, neutrophiles have several known effects in relation to cancer cells, one of which is the expulsion of NETs by activated neutrophils, as a response to trauma or inflammation [11]. Previous studies have shown that tumor cells adhere to, and form colonies on NETs, taking benefit from the anti-inflammatory effects of NETs, thus evading potentially tumor-depletory immune cells like T-cells and macrophages [10, 16]. A depletion of neutrophiles has in earlier studies been associated with a decrease in NET formation and consequently a statistically significant reduction in tumor growth [17–20]. This is one plausible theory behind a beneficial effect of depleted white blood cell counts on long term prognosis. As neutropenia does not develop until several days postoperatively following CRS and HIPEC, i.e., long after these events and effects occur, it might explain that no positive effect on recurrence rate or survival could be noted in the present study.

Another conceivable explanation to a relation between immune suppression after CRS and HIPEC and improved recurrence rate, is that leukopenia acts as a surrogate marker of cytotoxic effect, reflecting an effective HIPEC treatment that also leads to a diminished risk of recurrent disease. However, the results in the current study do not support this hypothesis.

The refinement of HIPEC treatment has generally been focused on maximizing the efficacy of the chemotherapeutic agent locally at the peritoneal surfaces, while minimizing the systemic toxicity [21–23]. Large molecular-weight substances have been preferred, with the intention of minimizing the systemic uptake due to the peritoneal-plasma barrier [24]. The combination treatment of oxaliplatin and irinotecan is known to be especially prone to cause myeloid dysfunction. In the event of chemotherapy-induced bone marrow depletion and subsequent leukopenia postoperatively, colony-stimulating factors (G-CSF) have been used routinely to counteract this condition, when established. Prophylactic treatment has also been used, but notably, during the time period when the combination treatment was used in Sweden, prophylactic bone marrow stimulation by G-CSF-treatment was not routine.

In the present study, 9,2% of the cases developed leukopenia (WBC < 1600/µL), which is in line with previous reports [25, 26], although the true figure for the whole study period is somewhat uncertain as the first time period had many missing data. Patient and tumor characteristics in this group did not differ statistically significant compared to the group without postoperative leukopenia. The most important factor associated with leukopenia in the current study was combination treatment with oxaliplatin and irinotecan. In these patients, leukopenia was observed in 46% compared to 6% among those receiving only oxaliplatin (*p* < 0.01). An increased risk of leukopenia related to the combined treatment has been reported earlier [27] and also a risk of bone marrow aplasia [28] leading to the gradual reduction of this treatment combination [29].

Leukopenia has been reported to be associated with an increased postoperative complication rate [30, 31] and postoperative complications have in turn been reported to be associated with a worse long-term prognosis [32–34]. Both findings were confirmed in this study. We found an overall complication rate of 74.1% in the leukopenia group compared to 57.1% in the non-leukopenia group (*p* = 0.016), and an increased risk of 3-year mortality after severe postoperative complication, HR 2.39 (95% CI: 1.39–4.13). This might be related to more extensive surgery in these cases, producing larger wound surfaces intra-abdominally, and a greater uptake of the cytotoxic substance, leading to systemic effects associated with increased risk of complications [35]. This hypothesis is supported by the finding in multivariate analysis, showing that prolonged duration of surgery wasassociated to more complications, and so was postoperative severe leukopenia.

This study has some limitations. As the Swedish national HIPEC registry’s variable for myelosuppression is leukopenia, in a 3-tier grading system, information on the actual levels of neutrophils is lacking. Moreover, most research on immunosuppression and effects on complications, metastases and long-term prognosis after oncologic treatment is based on neutrophil levels. Although neutrophils are the predominant part of white blood cell count, constituting about 50–70%, there is no absolute correlation between leukocyte and neutrophil levels. In the current study, leukopenia was used as a surrogate marker for neutropenia, with obvious limitations.

As in all registry-based research, the results are dependent on the completeness of the data in the registry. As seen in the tables, around five to six% of data are missing in most variables, which could be considered acceptable in clinical settings. However, the analysis of three-year recurrence-free survival had 11,8% missing values, which somewhat hampers the possibilities for conclusions in this category. Moreover, data on WBC was missing in the majority of cases in the first time period, making this group very small, and risk assessments for this time period unreliable. This is exemplified by a low risk of recurrence but not a low risk of mortality in this period. Another possible limitation is that the validity of data in the registry has not been evaluated and the relatively small group of patients with leukopenia hampers the reliability of subgroup analyses, such as on severe leukopenia (*n* = 32). Moreover, data on whether the patient received granulocyte colony stimulating factor (G-CSF) postoperatively or not was missing in a high rate (28 out of 54) and we lack data on the duration of leukopenia, making any analyses on these factors impossible.

The long study period, from 2003 to 2021, also implies some limitations, as treatment regimens and practices have changed over time following international trends, although neoadjuvant chemotherapy was not used routinely in Sweden during the study period. However, we perceive this as a minor problem as the study focuses on the effects of leukopenia, irrespective of the cause and time-periods were included in the multivariate analyses. Further, there might be a learning-curve effect, as the material includes all CRS and HIPEC procedures from the start in Sweden, reflected by some differences in outcome between different time periods. For example, a tendency towards higher risk of death within three years during the time period 2016–2018 compared to the most recent (Table 4). As time periods were included in the multivariate analyses, this bias was, at large, compensated for.

A strength of the current study is that it is population based, as all CRS and HIPEC cases in Sweden were included. We perceive that this fact contributes to making our findings valid in other clinical settings. Another strength is the prospectively registered data in the registry.

## Conclusion

The earlier finding that postoperative neutropenia could have an advantageous effect on long-term risk of recurrence after CRS and HIPEC for carcinomatosis of colorectal origin, was not verified in this study. On the contrary, there was a statistically non-significant tendency towards worse three-year survival in patients with severe postoperative leukopenia. HIPEC with the combination of oxaliplatin and irinotecan, was strongly associated with development of leukopenia.

## Electronic supplementary material

Below is the link to the electronic supplementary material.

Supplementary Material 1

## Data Availability

The original anonymous dataset is available on request from the corresponding author at mattias.lepsenyi@med.lu.se.
