# Supplementary material for: Postoperative leukopenia after cytoreductive surgery and hypertherm intraperitoneal chemotherapy for colorectal carcinomatosis– causes and implication on outcomes in a population-based study
Source: World J Surg Oncol. 2025 Apr 29;23:173. doi: 10.1186/s12957-025-03821-2 (PMC12042315; doi:10.1186/s12957-025-03821-2)
Supplement: Supplementary file 1 — Supplementary Material 1 [file 12957_2025_3821_MOESM1_ESM.docx]

Supplementary Fig. 1. Flow-chart on study recruitment.


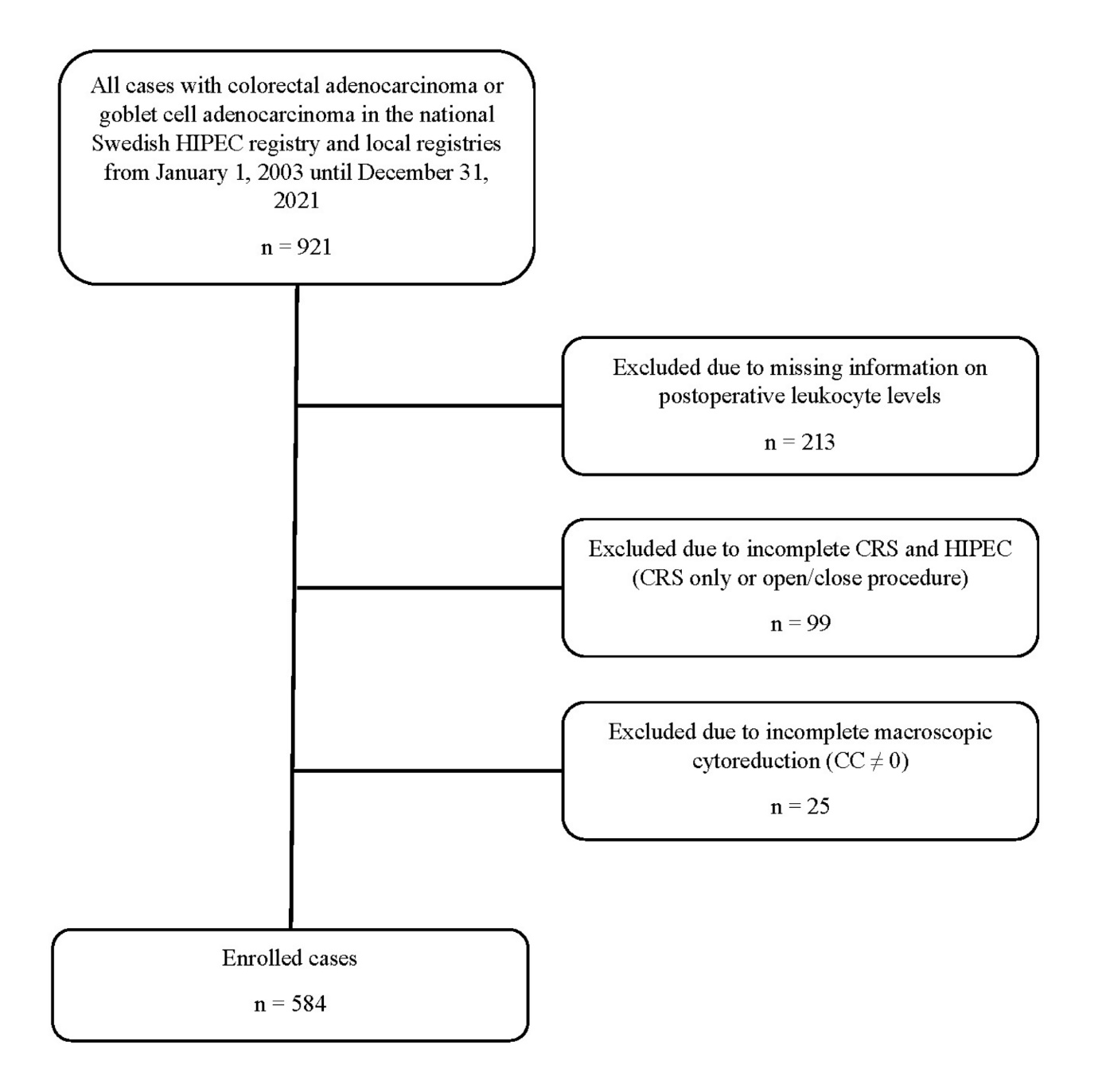



| Supplementary table 1. Risk of recurrence within 3 years* estimated by Cox proportional hazard ratio model. | | | | | | | | | | | | | | | | | | | | | | | | | | | |  |  |
| --- | --- | --- | --- | --- | --- | --- | --- | --- | --- | --- | --- | --- | --- | --- | --- | --- | --- | --- | --- | --- | --- | --- | --- | --- | --- | --- | --- | --- | --- |
|  | | Missing | | | | In analysis, | | | | **Univariate analysis** | | *p-*value | | | |  | | **Multivariate analysis** | | | | | | *p-*value | | | |  |  |
|  |  | data, n | | | | n | | | | HR (95% CI) | |  | | | |  |  | HR (95% CI) | | | | | |  | | | |  |  |
| **Age:** | | 48 | | | |  | | | |  | |  | | | |  | |  | | | | | |  | | | |  |  |
| < 65 years | |  | | | | 292 | | | | ref. | |  | | | |  | | ref. | | | | | |  | | | |  |  |
| ≥ 65 years | |  | | | | 244 | | | | 1.019 (0.829–1.253) | | 0.855 | | | |  | | 0.932 (0.728–1.193) | | | | | | 0.567 | | | |  |  |
| **Sex:** | | 46 | | | |  | | | |  | |  | | | |  | |  | | | | | |  | | | |  |  |
| Male | |  | | | | 246 | | | | 1.053 (0.857–1.294) | | 0.623 | | | |  | | 1.081 (0.844–1.384) | | | | | | 0.539 | | | |  |  |
| Female | |  | | | | 292 | | | | ref. | |  | | | |  | |  | | | | | |  | | | |  |  |
| **Neoadjuvant chemo:** | | 166 | | | |  | | | |  | |  | | | |  | |  | | | | | |  | | | |  |  |
| Yes | |  | | | | 125 | | | | 1.323 (1.029–1.701) | | 0.029 | | | |  | | 1.394 (1.073–1.811) | | | | | | 0.013 | | | |  |  |
| No | |  | | | | 293 | | | | ref. | |  | | | |  | |  | | | | | |  | | | |  |  |
| **Any complication:** | | 51 | | | |  | | | |  | |  | | | |  | |  | | | | | |  | | | |  |  |
| Yes | |  | | | | 305 | | | | 1.074 (0.870–1.324) | | 0.508 | | | |  | |  | | | | | |  | | | |  |  |
| No | |  | | | | 228 | | | | ref. | |  | | | |  | |  | | | | | |  | | | |  |  |
| **Severe complication:** | | 34 | | | |  | | | |  | |  | | | |  | |  | | | | | |  | | | |  |  |
| Yes | |  | | | | 68 | | | | 1.048 (0.755–1.455) | | 0.777 | | | |  | |  | | | | | |  | | | |  |  |
| No complication | |  | | | | 228 | | | | ref. | |  | | | |  | |  | | | | | |  | | | |  |  |
| Not in analysis (C-D 1-3a) | |  | | | | 254 | | | |  | |  | | | |  | |  | | | | | |  | | | |  |  |
| **Duration of surgery:** | | 64 | | | |  | | | |  | |  | | | |  | |  | | | | | |  | | | |  |  |
| < 480 min | |  | | | | 271 | | | | ref. | |  | | | |  | |  | | | | | |  | | | |  |  |
| ≥ 480 min | |  | | | | 249 | | | | 1.097 (0.890–1.351) | | 0.385 | | | |  | |  | | | | | |  | | | |  |  |
| **Postop leukopenia:** | | 44 | | | |  | | | |  | |  | | | |  | |  | | | | | |  | | | |  |  |
| Yes | |  | | | | 51 | | | | 0.962 (0.676–1.369) | | 0.83 | | | |  | | 0.831 (0.544–1.268) | | | | | | 0.389 | | | |  |  |
| No | |  | | | | 489 | | | | ref. | |  | | | |  | |  | | | | | |  | | | |  |  |
| **Severe leukopenia:** | | 43 | | | |  | | | |  | |  | | | |  | |  | | | | | |  | | | |  |  |
| Yes | |  | | | | 30 | | | | 1.050 (0.676–1.631) | | 0.829 | | | |  | |  | | | | | |  | | | |  |  |
| No | |  | | | | 489 | | | | ref. | |  | | | |  | |  | | | | | |  | | | |  |  |
| Not in analysis (mild leukopenia) | | | | | | 22 | | | |  | |  | | | |  | |  | | | | | |  | | | |  |  |
| **PCI-score:** | | 48 | | | |  | | | |  | |  | | | |  | |  | | | | | |  | | | |  |  |
| 0–8 | |  | | | | 265 | | | | ref. | |  | | | |  | | ref. | | | | | |  | | | |  |  |
| 9–15 | |  | | | | 157 | | | | 1.655 (1.302–2.104) | | < 0.001 | | | |  | | 1.463 (1.103–1.940) | | | | | | 0.008 | | | |  |  |
| > 15 | |  | | | | 114 | | | | 1.562 (1.205–2.025) | | < 0.001 | | | |  | | 1.538 (1.122–2.107) | | | | | | 0.007 | | | |  |  |
| **pN-stage:** | | 68 | | | |  | | | |  | |  | | | |  | |  | | | | | |  | | | |  |  |
| N0 | |  | | | | 106 | | | | ref. | |  | | | |  | | ref. | | | | | |  | | | |  |  |
| N1 | |  | | | | 183 | | | | 1.961 (1.422–2.705) | | < 0.001 | | | |  | | 1.855 (1.264–2.724) | | | | | | 0.002 | | | |  |  |
| N2 | |  | | | | 224 | | | | 2.239 (1.638–3.061) | | < 0.001 | | | |  | | 2.630 (1.809–3.822) | | | | | | < 0.001 | | | |  |  |
| Nx | |  | | | | 3 | | | |  | |  | | | |  | |  | | | | | |  | | | |  |  |
| **Period of surgery:** | | 44 | | | |  | | | |  | |  | | | |  | |  | | | | | |  | | | |  |  |
| 2019–2021 | |  | | | | 198 | | | | ref. | |  | | | |  | | ref. | | | | | |  | | | |  |  |
| 2016–2018 | |  | | | | 221 | | | | 0.875 (0.693–1.106) | | 0.264 | | | |  | | 0.964 (0.720–1.290) | | | | | | 0.803 | | | |  |  |
| 2013–2015 | |  | | | | 100 | | | | 0.731 (0.545–0.979) | | 0.036 | | | |  | | 0.719 (0.503–1.027) | | | | | | 0.069 | | | |  |  |
| ≤ 2012 | |  | | | | 21 | | | | 0.519 (0.264–1.021) | | 0.058 | | | |  | | 0.263 (0.095–0.733) | | | | | | 0.011 | | | |  |  |
| **Synchronous PC** | | 50 | | | |  | | | |  | |  | | | |  | |  | | | | | |  | | | |  |  |
| Yes | |  | | | | 347 | | | | 0.804 (0.650–0.994) | | 0.044 | | | |  | | 0.641 (0.498–0.826) | | | | | | < 0.001 | | | |  |  |
| No | |  | | | | 187 | | | | ref. | |  | | | |  | | ref. | | | | | |  | | | |  |  |
| * 90 day mortality excluded (n = 12) | |  | | | |  | | | |  | |  | | | |  | |  | | | | | |  | | | |  |  |
| Supplementary table 2. Risk of mortality within 3 years* estimated by Cox proportional hazard ratio model. | | | | | | | | | | | | | | | | | | | | | | | | | | | |  |  |
|  | | | Missing | | | | In analysis, | | | | **Univariate analysis** | | *p-*value | | | |  | | | | **Multivariate analysis** | | | | *p-*value | | |  |  |
|  | | | data, n | | | | n | | | | HR (95% CI) | |  | | | |  | | | | HR (95% CI) | | | |  | | |  |  |
| **Age:** | | | 43 | | | |  | | | |  | |  | | | |  | | | |  | | | |  | | |  |  |
| < 65 years | | |  | | | | 299 | | | | ref. | |  | | | |  | | | | ref. | | | |  | | |  |  |
| ≥ 65 years | | |  | | | | 242 | | | | 1.037 (0.773–1.391) | | 0.809 | | | |  | | | | 1.077 (0.754–1.541) | | | | 0.683 | | |  |  |
| **Sex:** | | | 42 | | | |  | | | |  | |  | | | |  | | | |  | | | |  | | |  |  |
| Male | | |  | | | | 249 | | | | 1.043 (0.777–1.399) | | 0.78 | | | |  | | | | 1.043 (0.729–1.491) | | | | 0.817 | | |  |  |
| Female | | |  | | | | 293 | | | | ref. | |  | | | |  | | | |  | | | |  | | |  |  |
| **Neoadjuvant chemo:** | | | 164 | | | |  | | | |  | |  | | | |  | | | |  | | | |  | | |  |  |
| Yes | | |  | | | | 127 | | | | 1.376 (0.978–1.934) | | 0.067 | | | |  | | | | 1.632 (1.123–2.372) | | | | 0.010 | | |  |  |
| No | | |  | | | | 293 | | | | ref. | |  | | | |  | | | |  | | | |  | | |  |  |
| **Any complication** **:** | | | 48 | | | |  | | | |  | |  | | | |  | | | |  | | | |  | | |  |  |
| Yes | | |  | | | | 311 | | | | 1.663 (1.205–2.295) | | 0,002 | | | |  | | | |  | | | |  | | |  |  |
| No | | |  | | | | 225 | | | | ref. | |  | | | |  | | | |  | | | |  | | |  |  |
| **Severe complication** **:** | | | 33 | | | |  | | | |  | |  | | | |  | | | |  | | | |  | | |  |  |
| Yes | | |  | | | | 72 | | | | 2.112 (1.373–3.249) | | < 0.001 | | | |  | | | | 1.821 (1.150–2.883) | | | | 0.011 | | |  |  |
| No | | |  | | | | 225 | | | | ref. | |  | | | |  | | | |  | | | |  | | |  |  |
| Not in analysis (CD 1-3a) | | |  | | | | 254 | | | |  | |  | | | |  | | | |  | | | |  | | |  |  |
| **Duration of surgery:** | | | 60 | | | |  | | | |  | |  | | | |  | | | |  | | | |  | | |  |  |
| < 480 min | | |  | | | | 267 | | | | ref. | |  | | | |  | | | |  | | | |  | | |  |  |
| ≥ 480 min | | |  | | | | 257 | | | | 1.313 (0.975–1.769) | | 0.073 | | | |  | | | | 0.897 (0.617–1.303) | | | | 0.568 | | |  |  |
| **Postop leukopenia:** | | | 40 | | | |  | | | |  | |  | | | |  | | | |  | | | |  | | |  |  |
| Yes | | |  | | | | 47 | | | | 0.877 (0.508–1.514) | | 0.638 | | | |  | | | |  | | | |  | | |  |  |
| No | | |  | | | | 497 | | | | ref. | |  | | | |  | | | |  | | | |  | | |  |  |
| **Severe leukopenia:** | | | 38 | | | |  | | | |  | |  | | | |  | | | |  | | | |  | | |  |  |
| Yes | | |  | | | | 27 | | | | 1.678 (0.934–3.015) | | 0.083 | | | |  | | | | 1.254 (0.576–2.731) | | | | 0.568 | | |  |  |
| No | | |  | | | | 497 | | | | ref. | |  | | | |  | | | |  | | | |  | | |  |  |
| Not in analysis (mild leukopenia) | | |  | | | | 22 | | | |  | |  | | | |  | | | |  | | | |  | | |  |  |
| **PCI-score:** | | | 45 | | | |  | | | |  | |  | | | |  | | | |  | | | |  | | |  |  |
| 0–8 | | |  | | | | 265 | | | | ref. | |  | | | |  | | | | ref. | | | |  | | |  |  |
| 9–15 | | |  | | | | 159 | | | | 2.084 (1.464–2.965) | | < 0.001 | | | |  | | | | 1.880 (1.236–2.860) | | | | 0.003 | | |  |  |
| > 15 | | |  | | | | 115 | | | | 2.632 (1.822–3.801) | | < 0.001 | | | |  | | | | 2.173 (1.357–3.435) | | | | < 0.001 | | |  |  |
| **pN-stage:** | | | 66 | | | |  | | | |  | |  | | | |  | | | |  | | | |  | | |  |  |
| N0 | | |  | | | | 107 | | | | ref. | |  | | | |  | | | | ref. | | | |  | | |  |  |
| N1 | | |  | | | | 181 | | | | 1.902 (1.118–3.234) | | 0.018 | | | |  | | | | 1.380 (0.753–2.526) | | | | 0.297 | | |  |  |
| N2 | | |  | | | | 227 | | | | 2.991 (1.809–4.944) | | < 0.001 | | | |  | | | | 2.329 (1.325–4.094) | | | | 0.003 | | |  |  |
| Nx | | |  | | | | 3 | | | |  | |  | | | |  | | | |  | | | |  | | |  |  |
| **Period of surgery:** | | | 40 | | | |  | | | |  | |  | | | |  | | | |  | | | |  | | |  |  |
| 2019–2021 | | |  | | | | 201 | | | | ref. | |  | | | |  | | | | ref. | | | |  | | |  |  |
| 2016–2018 | | |  | | | | 213 | | | | 1.546 (1.070–2.234) | | 0.02 | | | |  | | | | 1.875 (1.124–3.129) | | | | 0.016 | | |  |  |
| 2013–2015 | | |  | | | | 109 | | | | 1.541 (1.008–2.355) | | 0.046 | | | |  | | | | 1.630 (0.927–2.865) | | | | 0.090 | | |  |  |
| ≤ 2012 | | |  | | | | 21 | | | | 1.425 (0.669–3.034) | | 0.358 | | | |  | | | | 1.127 (0.398–3.195) | | | | 0.822 | | |  |  |
| **Synchronous PC** | | | 66 | | | |  | | | |  | |  | | | |  | | | |  | | | |  | | |  |  |
| Yes | | | |  | | | | 340 | 1.058 (0.775–1.445) | | | | | | 0.721 | | | |  | | | 0.801 (0.542–1.184) | | | | 0.266 | | |  |
| No | | | |  | | | | 178 | ref. | | | | | |  | | | |  | | | ref. | | | |  | | |  |
| * 90 day mortality excluded (n = 12) | | | |  | | | |  |  | | | | | |  | | | |  | | |  | | | |  | | |  |
| Supplementary table 3. Risk of postoperative leukopenia, estimated by logistic regression. | | | | | | | | | | | | | | | | | | | | | | | | | | | | | |
|  | Missing | | | | In analysis, | | | | **Univariate analysis** | | | | | *p-*value | | | | | |  | | | **Multivariate analysis** | | | | *p-*value | | |
|  | data, n | | | | n | | | | HR (95% CI) | | | | |  | | | | | |  |  |  | HR (95% CI) | | | |  | | |
| **Age:** | 4 | | | |  | | | |  | | | | |  | | | | | |  | | |  | | | |  | | |
| < 65 years |  | | | | 311 | | | | ref. | | | | |  | | | | | |  | | |  | | | |  | | |
| ≥ 65 years |  | | | | 269 | | | | 0.996 (0.568–1.747) | | | | | 0.99 | | | | | |  | | |  | | | |  | | |
| **Sex:** | 2 | | | |  | | | |  | | | | |  | | | | | |  | | |  | | | |  | | |
| Male |  | | | | 263 | | | | 0.819 (0.463–1.447) | | | | | 0.491 | | | | | |  | | |  | | | |  | | |
| Female |  | | | | 319 | | | | ref. | | | | |  | | | | | |  | | |  | | | |  | | |
| **Neoadjuvant chemo:** | 131 | | | |  | | | |  | | | | |  | | | | | |  | | |  | | | |  | | |
| Yes |  | | | | 136 | | | | 1.231 (0.637–2.378) | | | | | 0.536 | | | | | |  | | |  | | | |  | | |
| No |  | | | | 317 | | | | ref. | | | | |  | | | | | |  | | |  | | | |  | | |
| **Chemotherapy IP:** | 48 | | | |  | | | |  | | | | |  | | | | | |  | | |  | | | |  | | |
| Oxaliplatin |  | | | | 395 | | | | ref. | | | | |  | | | | | |  | | | ref. | | | |  | | |
| Oxaliplatin + Irinotecan |  | | | | 37 | | | | 13.140 (6.101–28.297) | | | | | < 0.001 | | | | | |  | | | 12.336 (4.510-33.739) | | | | < 0,001 | | |
| Mitomycin C |  | | | | 29 | | | | 3.220 (1.129–9.187) | | | | | 0.029 | | | | | |  | | | 3.003 (1.020–8.843) | | | | 0.046 | | |
| Irinotecan |  | | | | 70 | | | | 1.189 (0.438–2.229) | | | | | 0.734 | | | | | |  | | | 1.740 (0.613–4.939) | | | | 0.298 | | |
| Other |  | | | | 5 | | | | - | | | | |  | | | | | |  | | |  | | | |  | | |
| **Any complication:** | 8 | | | |  | | | |  | | | | |  | | | | | |  | | |  | | | |  | | |
| Yes |  | | | | 338 | | | | 2.148 (1.141–4.044) | | | | | 0.018 | | | | | |  | | | 1.977 (0.992–3.939) | | | | 0.053 | | |
| No |  | | | | 238 | | | | ref. | | | | |  | | | | | |  | | |  | | | |  | | |
| **Severe complication:** | 8 | | | |  | | | |  | | | | |  | | | | | |  | | |  | | | |  | | |
| Yes |  | | | | 82 | | | | 2.222 (0.946–5.219) | | | | | 0.067 | | | | | |  | | |  | | | |  | | |
| No |  | | | | 238 | | | | ref. | | | | |  | | | | | |  | | |  | | | |  | | |
| Not in analysis, (CD 1 ≤ 3a) |  | | | | 254 | | | |  | | | | |  | | | | | |  | | |  | | | |  | | |
| **Duration of surgery:** | 22 | | | |  | | | |  | | | | |  | | | | | |  | | |  | | | |  | | |
| < 480 min |  | | | | 281 | | | | ref. | | | | |  | | | | | |  | | |  | | | |  | | |
| ≥ 480 min |  | | | | 281 | | | | 2.147 (1.188–3.881) | | | | | 0.011 | | | | | |  | | | 1.536 (0.768–3.072) | | | | 0.225 | | |
| **PCI-score:** | 6 | | | |  | | | |  | | | | |  | | | | | |  | | |  | | | |  | | |
| 0–8 |  | | | | 279 | | | | ref. | | | | |  | | | | | |  | | |  | | | |  | | |
| 9–15 |  | | | | 172 | | | | 0.796 (0.397–1.594) | | | | | 0.519 | | | | | |  | | |  | | | |  | | |
| > 15 |  | | | | 127 | | | | 1.206 (0.607–2.395) | | | | | 0.594 | | | | | |  | | |  | | | |  | | |
| **Period of surgery:** | 0 | | | |  | | | |  | | | | |  | | | | | |  | | |  | | | |  | | |
| 2019–2021 |  | | | | 218 | | | | ref. | | | | |  | | | | | |  | | | ref. | | | |  | | |
| 2016–2018 |  | | | | 233 | | | | 1.437 (0.675–3.058) | | | | | 0.346 | | | | | |  | | | 1.647 (0.733–3.701) | | | | 0.228 | | |
| 2013–2015 |  | | | | 112 | | | | 3.287 (1.522-7.100) | | | | | 0.002 | | | | | |  | | | 1.357 (0.512-3.600) | | | | 0.539 | | |
| ≤ 2012 |  | | | | 21 | | | | 6.867 (2.260-20.864) | | | | | < 0.001 | | | | | |  | | | 1.213 (0.294–4.998) | | | | 0.789 | | |

| Supplementary table 4. Risk of any postoperative complication, estimated by logistic regression. | | | | | | | |
| --- | --- | --- | --- | --- | --- | --- | --- |
|  | Missing | In analysis, | **Univariate analysis** | *p-*value |  | **Multivariate analysis** | *p-*value |
|  | data, n | n | HR (95% CI) |  |  | HR (95% CI) |  |
| **Age:** | 11 |  |  |  |  |  |  |
| < 65 years |  | 304 | ref. |  |  |  |  |
| ≥ 65 years |  | 269 | 1.134 (0.812–1.583) | 0.462 |  |  |  |
| **Sex:** |  |  |  |  |  |  |  |
| Male |  | 256 | 1.128 (0.807–1.577) | 0.48 |  |  |  |
| Female |  | 318 | ref. |  |  |  |  |
| Missing data |  | 10 | - |  |  |  |  |
| **Neoadjuvant chemo:** | 136 |  |  |  |  |  |  |
| Yes |  | 134 | 1.171 (0.773–1.776) | 0.456 |  |  |  |
| No |  | 314 | ref. |  |  |  |  |
| **Chemotherapy IP:** | 54 |  |  |  |  |  |  |
| Oxaliplatin |  | 390 | ref. |  |  | ref. |  |
| Oxaliplatin + Irinotecan |  | 37 | 1.627 (0.794–3.331) | 0.183 |  | 0.913 (0.369–2.258) | 0.844 |
| Mitomycin C |  | 29 | 0.961 (0.450–2.052) | 0.918 |  | 0.897 (0.386–2.084) | 0.8 |
| Irinotecan |  | 68 | 0.547 (0.324–0.922) | 0.024 |  | 0.696 (0.392–1.236) | 0.216 |
| Other |  | 6 | - |  |  |  |  |
| **Duration of surgery:** | 30 |  |  |  |  |  |  |
| < 480 min |  | 277 | ref. |  |  |  |  |
| ≥ 480 min |  | 277 | 2.459 (1.735–3.484) | < 0.001 |  | 2.299 (1.545–3.422) | < 0,001 |
| **PCI-score:** | 14 |  |  |  |  |  |  |
| 0–8 |  | 274 | ref. |  |  | ref. |  |
| 9–15 |  | 171 | 1.100 (0.748–1.616) | 0.628 |  | 0.916 (0.596–1.408) | 0.69 |
| > 15 |  | 125 | 1.921 (1.226–3.010) | 0.004 |  | 1.323 (0.790–2.215) | 0.287 |
| **Postop leukopenia:** | 8 |  |  |  |  |  |  |
| Yes |  | 54 | 2.148 (1.141–4.044) | 0.018 |  |  |  |
| No |  | 522 | ref. |  |  |  |  |
| **Severe leukopenia:** | 8 |  |  |  |  |  |  |
| Yes |  | 32 | 4.059 (1.539–10.706) | 0.005 |  | 3.495 (1.251–9.765) | 0.017 |
| No |  | 522 | ref. |  |  |  |  |
| Not in analysis (mild leukopenia) | | 22 |  |  |  |  |  |
| **Period of surgery:** | 8 |  |  |  |  |  |  |
| 2019–2021 |  | 213 | ref. |  |  | ref. |  |
| 2016–2018 |  | 232 | 1.847 (1.262–2.701) | 0.002 |  | 1.027 (0.669–1.578) | 0.902 |
| 2013–2015 |  | 111 | 1.596 (1.001–2.546) | 0.05 |  | 1.062 (0.618–1.823) | 0.828 |
| ≤ 2012 |  | 20 | 2.355 (0.872–6.360) | 0.091 |  | 2.161 (0.666–7.017) | 0.2 |

| Supplementary table 5. Risk of severe postoperative complication, C-D > 3a, estimated by logistic regression. | | | | | | | |
| --- | --- | --- | --- | --- | --- | --- | --- |
|  | Missing | In analysis, | **Univariate analysis** | *p-*value |  | **Multivariate analysis** | *p-*value |
|  | data, n | n | HR (95% CI) |  |  | HR (95% CI) |  |
| **Age:** | 265 |  |  |  |  |  |  |
| < 65 years |  | 168 | ref. |  |  |  |  |
| ≥ 65 years |  | 151 | 1.404 (0.852–2.315) | 0.183 |  | 1.568 (0.871–2.825) | 0.134 |
| **Sex:** | 263 |  |  |  |  |  |  |
| Male |  | 146 | 1.504 (0.911–2.484) | 0.111 |  | 1.298 (0.723–2.329) | 0.383 |
| Female |  | 175 | ref. |  |  |  |  |
| **Neoadjuvant chemo:** | 340 |  |  |  |  |  |  |
| Yes |  | 72 | 1.320 (0.718–2.426) | 0.371 |  |  |  |
| No |  | 172 | ref. |  |  |  |  |
| **Chemotherapy IP:** | 271 |  |  |  |  |  |  |
| Oxaliplatin |  | 230 | ref. |  |  |  |  |
| Oxaliplatin + Irinotecan |  | 19 | 1.691 (0.636–4.496) | 0.293 |  | 1.251 (0.401–3.904) | 0.7 |
| Mitomycin C |  | 17 | 0.892 (0.280–2.842) | 0.846 |  | 1.046 (0.306–3.579) | 0.943 |
| Irinotecan |  | 44 | 0.290 (0.099–0.845) | 0,023 |  | 0.269 (0.076–0.954) | 0.042 |
| Other |  | 3 | - |  |  |  |  |
| **Duration of surgery:** | 277 |  |  |  |  |  |  |
| < 480 min |  | 174 | ref. |  |  |  |  |
| ≥ 480 min |  | 133 | 2.673 (1.590–4.496) | < 0.001 |  | 2.047 (1.115–3.760) | 0.021 |
| **PCI-score:** | 266 |  |  |  |  |  |  |
| 0–8 |  | 159 | ref. |  |  |  |  |
| 8–15 |  | 97 | 1.143 (0.626–2.087) | 0.664 |  | 0.694 (0.342–1.408) | 0.311 |
| > 15 |  | 62 | 2.322 (1.229–4.387) | 0,009 |  | 1.392 (0.655–2.959) | 0.39 |
| **Postop leukopenia:** | 262 |  |  |  |  |  |  |
| Yes |  | 24 | 2.162 (0.921–5.074) | 0.076 |  |  |  |
| No |  | 298 | ref. |  |  |  |  |
| **Severe leukopenia:** | 250 |  |  |  |  |  |  |
| Yes |  | 14 | 5.449 (1.770-16.772) | 0,003 |  | 4.312 (1.247–14.902) | 0.021 |
| No |  | 298 | ref. |  |  |  |  |
| Not in analysis (mild leukopenia) |  | 22 |  |  |  |  |  |
| **Period of surgery:** | 262 |  |  |  |  |  |  |
| 2019–2021 |  | 139 | ref. |  |  |  |  |
| 2016–2018 |  | 116 | 1.389 (0.787–2.452) | 0.257 |  |  |  |
| 2013–2015 |  | 58 | 1.204 (0.591–2.451) | 0.609 |  |  |  |
| ≤ 2012 |  | 9 | 1.726 (0.408–7.302) | 0.458 |  |  |  |
